# A Novel Mutation (D395A) in Valosin-Containing Protein Gene Is Associated With Early Onset Frontotemporal Dementia in an Italian Family

**DOI:** 10.3389/fgene.2021.795029

**Published:** 2021-11-30

**Authors:** Francesco Bruno, Maria Elena Conidi, Gianfranco Puccio, Francesca Frangipane, Valentina Laganà, Livia Bernardi, Nicoletta Smirne, Maria Mirabelli, Rosanna Colao, Sabrina Curcio, Raffaele Di Lorenzo, Raffaele Maletta, Amalia Cecilia Bruni

**Affiliations:** ^1^ Regional Neurogenetic Centre (CRN), Department of Primary Care, ASP Catanzaro, Lamezia Terme, Italy; ^2^ Laboratorio Analisi Dell'Ospedale G. Jazzolino—ASP Vibo Valentia (RC), Reggio Calabria, Italy

**Keywords:** *VCP* gene, novel mutation, D395A, frontotemporal dementia, bvFTD, body myopathy, Paget’s disease of bone, Valosin-Containing Protein

## Abstract

Inclusion body myopathy (IBM) with Paget’s disease of bone (PDB) and/or frontotemporal dementia (FTD) (IBMPFD) was recently identified as rare autosomal dominant disorder due to mutations in *VCP* gene. However, *VCP* mutations have also been documented in patients with amyotrophic lateral sclerosis (ALS), Charcot-Marie-Tooth type 2 (CMT2) disease, and hereditary spastic paraplegia (HSP), underlining the heterogeneity of the phenotypes due to *VCP* mutations. In this study, we reported a novel missense heterozygous variant c.1184A > C (*p*.D395A) in exon 10 of *VCP* gene identified in three patients (two sisters and one brother) belonging to an Italian family. The patients underwent a detailed clinical evaluation including medical history, neurological examination, and neuropsychological assessment. Brain’s morphologic and functional analysis was also performed. The whole picture was consistent with the criteria of behavioral variant frontotemporal dementia (bvFTD) without IBM and PBD. Our report confirms the high degree of heterogeneity of *VCP* disease. A *VCP* analysis should be considered for the genetic screening of familial bvFTD with an early onset also in absence of IBM or PDB signs.

## Introduction

The valosin-containing protein (*VCP*) gene is located on chromosome 9p13.3-p12 and encodes for VCP—also known as p97—a ubiquitously, essential, and multifunctional protein highly conserved in evolution (Papadimas et al., 2017; Lyupina et al., 2018). This protein includes different functional domains: two ATPase domains (D1 and D2), two linker domains (L1 and L2), an N-terminal ubiquitin–binding domain, and a C-terminal domain (Peters et al., 1990; Van den Boom and Meyer, 2018). It has been shown that VCP protein is a member of the AAA-ATPase superfamily, a group of chaperone-like proteins involved in multiple cellular processes including cell cycle control, membrane fusion, nuclear envelope reconstruction, post-mitotic Golgi reassembly, DNA damage response, suppressor of apoptosis, and ubiquitin-dependent protein degradation (Watts et al., 2004; Meyer & Weihl, 2014).

Currently, more than 45 *VCP* mutations have been identified (Saracino et al., 2018) as responsible of a rare multisystem proteinopathy known as inclusion body myopathy (IBM) associated with Paget’s disease of bone (PDB) and early onset frontotemporal dementia (FTD) (IBMPFD) (Mehta et al., 2013; Watts et al., 2004). *VCP*’s role in protein degradation and autophagy is probably implicated in the pathogenesis of IBMPFD and may account for the cytoplasmic inclusions observed in muscle, bone, and neuronal tissue (Al-Obeidi et al., 2018; Kimonis et al., 2008). The main clinical features of this disease may be found alone or in combination in affected individuals (Al-Obeidi et al., 2018). Most of the *VCP* mutation carriers have a positive family history with a great phenotypic heterogeneity within and between families (Nakamura et al., 2021; Papadimas et al., 2017). Because of this lack of genotype–phenotype correlations, *VCP*-related disease may be considered as an autosomal dominant inherited spectrum of disorders with incomplete penetrance (Papadimas et al., 2017). Indeed, *VCP* mutations have also been reported in patients with amyotrophic lateral sclerosis (ALS) (Matsubara et al., 2021), Charcot-Marie-Tooth type 2 (CMT2) disease (Gonzalez et al., 2014; Gite et al., 2020), and hereditary spastic paraplegia (HSP) (van de Warrenburg et al., 2016). Moreover, Saracino et al. (2018) reported a *VCP*-mutated patient with FTD that did not develop clinical symptoms of PDB or IBM (Saracino et al., 2018). Here, we have described three Italian siblings carrying a novel *VCP* mutation (D395A) who were affected by early-onset behavioral variant frontotemporal dementia (bvFTD). No PDB and IBM signs were clinically observed. To our knowledge, this is the first description of *VCP*-related bvFTD phenotype in Italian patients belonging to the same family.

## Patients and Methods

### Patients

The patients underwent a detailed clinical evaluation including medical history, neurological examination (including inspection, strength, deep tendon reflexes, plantar responses, and sensory examination), and neuropsychological assessment (i.e., Mini-Mental State Examination, MMSE; Clinical Dementia Rating Scale, CDR). Brain morphologic and functional imaging including computed tomography (CT), magnetic resonance imaging (MRI), and single photo emission computed tomography (SPECT) was reviewed for the three patients to confirm the diagnosis and to rule out any potential alternative diagnosis. Routine blood tests were performed including serum measurements of creatine kinase (CK) and alkaline phosphatase (ALP). The final diagnosis was established fulfilling the current clinical criteria for bvFTD (Rascovsky et al., 2011). Moreover, 150 ethnically matched healthy subjects were recruited and carefully assessed by using a rigorous clinical history evaluation and a general and neuropsychological examination to exclude any neurological disorder. Informed consent for the study was obtained directly from patients or, when appropriate, from relative or legal guardians. The study was conducted according to Helsinki Declaration of 1975 and approved by the Ethical Committee of Calabria Region (Catanzaro, Italy).

### Molecular Screening

Genomic DNA of the three patients was extracted from peripheral blood lymphocytes using standard methods. The proband was analyzed through the use of the Ion Torrent PGM sequencer (Thermo Fisher Scientific, Waltham, MA United States). An AmpliSeqTM custom panel (Thermo Fisher Scientific, Waltham, MA United States) of 16 known FTD-related genes (*GRN, MAPT, TREM2, CHMP2B, CSF1R, FUS, TBK1, VCP, TARDBP, SQSTM1, DCTN1, CHCHD10, PRNP, SOD1, PS1,* and *PS2*) was designed. All variants were classified as rare variant or mutation if their frequency is lower than 1% in at least one of the three reference databases (The Human Gene Mutation Database at the Institute of Medical Genetics in Cardiff, http://www.hgmd.org; the 1,000 Genomes Project http://www.internationalgenome.org/; the Exome Sequencing Project http://evs.gs.washington.edu/EVS/;and the Exome Aggregation Consortium http://308 exac.broadinstitute.org) and then compared with literature data to define whether they are previously reported or novel variants. Validation of the NGS findings in proband and the segregation analysis of the new variant in two affected sibs was performed by Sanger sequencing of *VCP* exon 10. The C9orf72 hexanucleotide (G4C2) repeat expansion mutation was determined using a repeat-primed PCR analysis. Moreover, 150 healthy subjects were analyzed to exclude a common polymorphism using a PCR and restriction fragment length polymorphism method (RFLP) for base change in exon 10 using HaeIII restriction enzyme according to manufacturer’s protocol (Promega, United States). *In silico* predictin software (PolyPhen-2 (Prediction of Functional Effects of Human nsSNPs, http://genetics.bwh.harvard.edu/pph2/), SIFT (Sorting Intolerant from Tolerant, http://sift.jcvi.org/), MutationTaster (http://www.mutationtaster.org), Provean Protein (http://provean.jcvi.org/seq_submit.php), and FATHMM (http://fathmm.biocompute.org.uk/) were used to assess the effect of this new variant. The Varsome program (https://varsome.com/) was also applied to describe the variants. Sequence cluster alignment was performed using Clustal Omega to compare related sequences from different species.

## Results

### Case Reports

**FIGURE 1:**
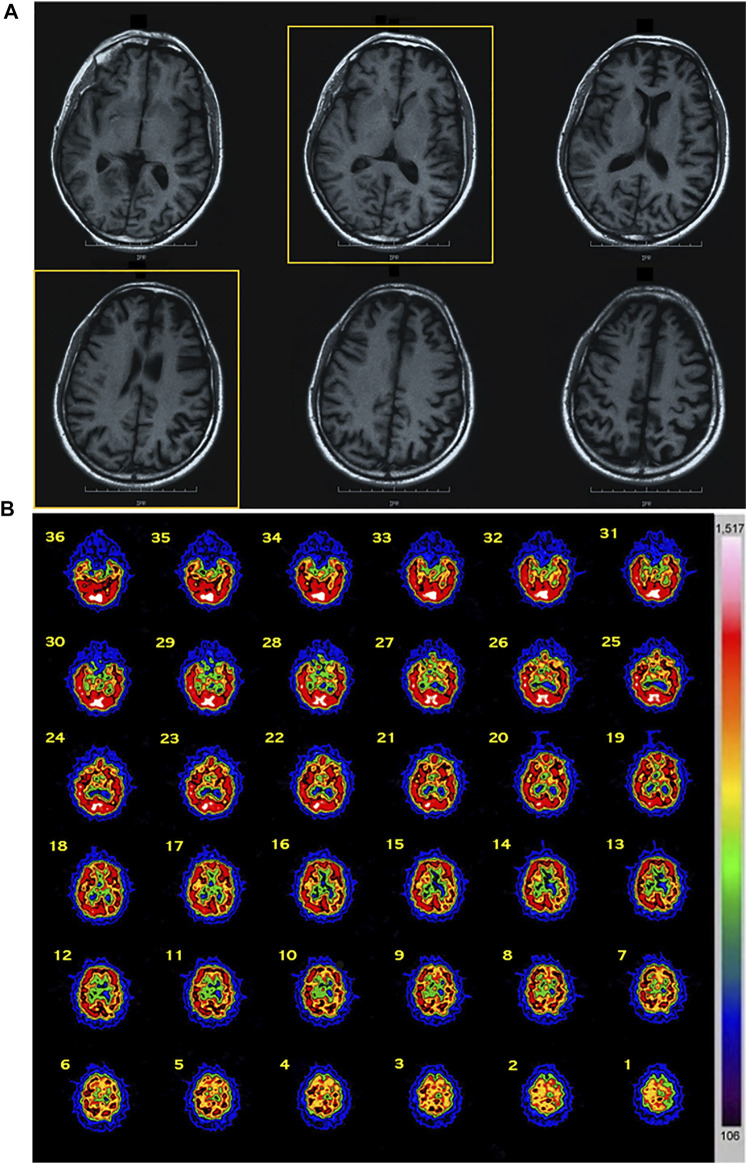
Imaging data of the proband. **(A)** MRI data: diffuse cortical atrophy prominent in frontal and temporal regions was present. **(B)** SPECT data: a hypoperfusion was observed in frontal and temporal convolutions of the left hemisphere and upper frontal circumvolution of the right hemisphere. Abbreviations: MRI, magnetic resonance imaging; SPECT, single-photon emission computed tomography.

Patient 1 (II-3): The proband was a 40-year-old male. From the age of 35 years, the patient developed progressive behavior abnormalities including apathy, social withdrawal, emotional blunting delusions, agitation, and aggression. During the following months, he presented progressive inattention and memory impairment with loss of personal hygiene habits. EEG, performed at the age of 38 years, manifested an angular aspect delta lens waves diffused with left frontotemporal prevalence. At 40 years old, he was evaluated at our center because of progressive deterioration of criticism and judgment, abilities, stereotypies, and ritualistic behaviors (he picked up cigarette butts on the streets and tried to smoke them), and he presented lack of disease insight. Neurological examination revealed ideomotor apraxia and rooting reflex. Neuropsychological evaluation showed a widespread severe impairment of cognitive functions with predominant involvement of frontal lobe functions. His score on MMSE was 14.9/30 and 2 on the CDR scale. Afterward, about 3 years after his first observation, bulimia (eats uncooked foods), visual hallucinations, hetero-aggressiveness (Neuropsychiatric Inventory, NPI = 68), hyperorality, hypersexuality, manifestations of self-harm, and almost absent language appeared together with generalized epileptic seizures. At 46 years old, in the end stage of his disease, cognitive deterioration was severe with prominent executive dysfunction and marked behavioral disturbances, and finally he showed a severe frontal dementia. He became uncommunicative, rigid, incontinent, and loss of ability to perform activities of daily living (Activities of Day Living, ADL = 2; Instrumental Activities of Daily Living, IADL = 1). He died at 47 years because of intercurrent infections. Routine blood examinations including serum creatine phosphokinase (CPK) and alkaline phosphatase (AP) revealed no abnormalities. Brain MRI showed diffuse cortical atrophy prominent in frontal and temporal regions (Figure 1A). Brain SPECT confirmed these findings revealing a hypoperfusion in frontal and temporal convolutions of the left hemisphere and upper frontal circumvolution of the right hemisphere (Figure 1B).

Patient 2 (II-1): the 45-year-old sister presented a clinical picture quite similar to the index patient at about 43 years. Over time, a gradual cognitive impairment together with progressive behavioral changes and unawareness became evident. She had neglect for personal hygiene, foul language, and delusions with verbal and physical aggressiveness. At first clinical examination, she showed emotional flatness with criticism and judgment reductions together with behavioral alterations. Cognitive impairment occurred right after with reduced working memory and attention impairment and difficulties in abstract reasoning, planning, and problem solving. Her score on MMSE was 19.9/30 and 1 on the CDR scale. In addition, she showed a high degree of dependence for activities of daily living (ADL = 3; IADL = 1). During the following years, a clear FTD picture with recurrent environmental dependency behaviors (i.e., she ate paper napkins waiting for the meal) was evident. At 46 years, she manifested generalized epileptic seizures. Neuropsychological evaluation confirmed a prominent impairment of executive functions, abstract reasoning, and judgment abilities associated with environmental dependency (MMSE = 17,9). Neurological examination revealed ideomotor apraxia and rooting reflex. Routine blood examinations including serum creatine phosphokinase (CPK) and alkaline phosphatase (AP) revealed no abnormalities. CT scan showed diffuse cortical atrophy prominent in the frontal lobes and brain SPECT disclosed bilateral fronto–parieto–temporal hypoperfusion.

Patient 3 (II-2): The patient was a 44-year-old woman (sister of proband and patient 2); 2 years before she became apathetic, ineffective, emotionally blunted with respect to some important tragic family events. At 43 years old, the patient manifested generalized epileptic seizures during sleep, bulimia, motor stereotypies such as “body rocking,” and progressive loss of language with vocalizations. In clinical evaluation, she showed severe memory impairment, alterations of spatial orientation, and later developed progressive loss of personal care. At first assessment, her score on the Mini-Mental Status Examination was 10.9/30 and 1 on the CDR Scale. She was unable to perform most activities of daily living (ADL = 3 and IADL = 3). The neurological examination, also in this case, highlighted ideomotor apraxia and rooting reflex. Laboratory examinations including CPK and AP were unremarkable. Brain CT scan showed diffuse cortical atrophy and SPECT disclosed bilateral fronto–parieto–temporal hypoperfusion. Diffused theta waves were appreciated at the EEG.

### Family History

**FIGURE 2:**
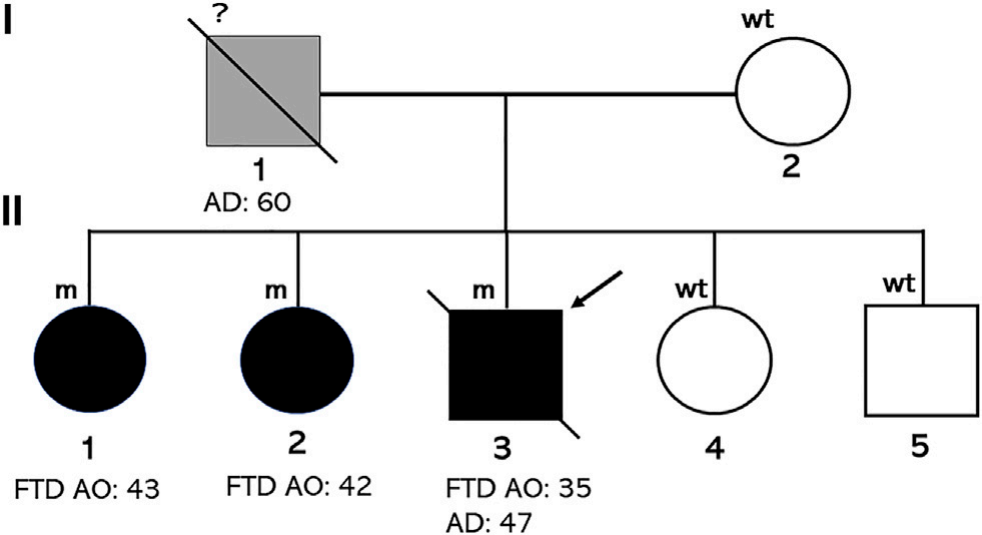
Pedigree of the family. Individuals affected by FTD carrying the D395A mutation are indicated with black diamonds (male) or circle (females). The arrow indicates the proband. Age at onset (AO) or at death (AD) is indicated.

Family history of these three sibs was not significant about neurodegenerative diseases, although their father died at 60 years due to a referred fall, after spending last years of his life in a psychiatric hospital (Figure 2).

### Genetics Findings

**FIGURE 3:**
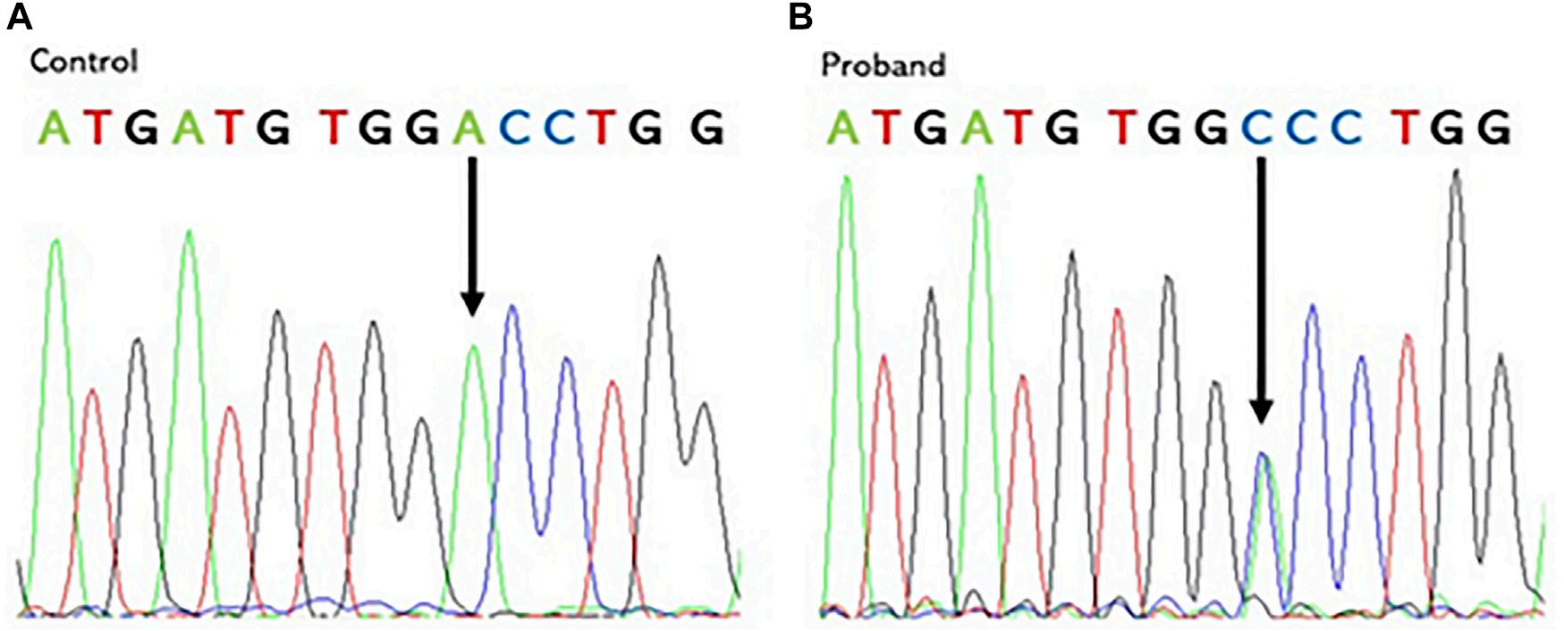
Chromatogram of the control **(A)** and the proband **(B)** showing the heterozygous *VCP* mutation: a c.1184A > C nucleotide substitution in exon 10 at codon 395. Vertical arrows indicate the mutation site.

PGM sequencing of the proband revealed, at the heterozygote state, a novel variant c.1184A > C in the exon 10 of *VCP* (Refseq ID: NM_126007.5). This substitution results in an Aspartate to an Alanine change at codon 395 (*p*.D395A), shifting this residue from polar hydrophilic to non-polar and hydrophobic. The presence of this variant was confirmed in the proband and in his two affected sisters using Sanger sequencing (Figure 3).

**FIGURE 4:**
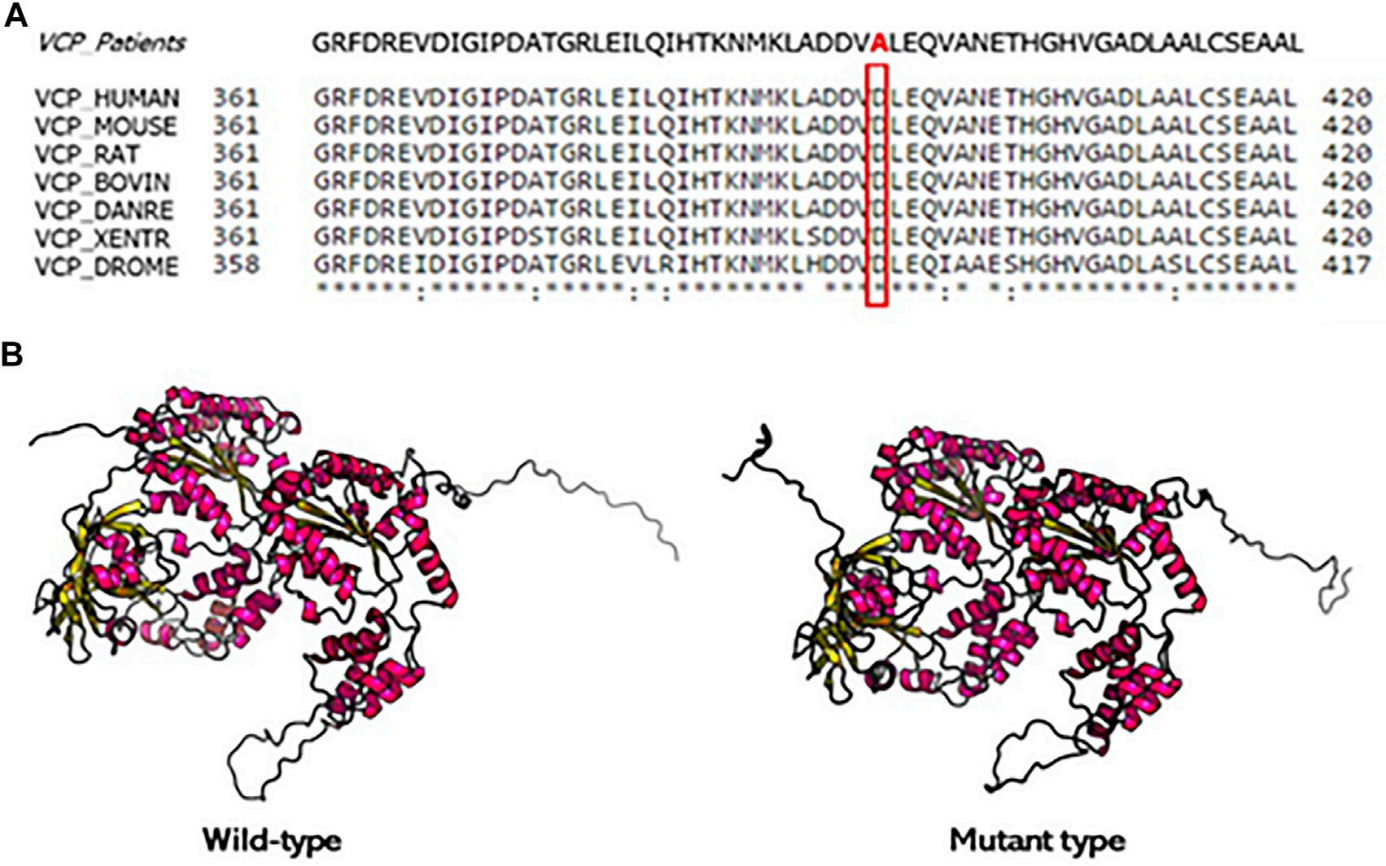
Conservation analysis of the mutation site and protein structure modeling of VCP. **(A)** Alignment of *VCP* sequences from different species. Mutated residue is boxed. **(B)** The 3D protein structure modeling wild type and mutation type of VCP protein. The D395A mutation changes the residue side chain at the positions 395 of VCP protein.

We did not find any pathogenic variant in the other examined genes. The patients have a possible family history of disease since their father died in a psychiatric hospital but, unfortunately, the segregation study could not be performed because DNA of other relatives was not available. The D395A variant is not detected in our healthy controls and absent from public databases of DNA controls. Moreover, the new variant has been bioinformatically analyzed on Varsome and classified as “likely pathogenetic” according to the American College of Medical Genetics (ACMG) criteria (Richards et al., 2015). PM2) D395A VCP variant has not been found in gnomAD exomes; PM5) an alternative amino acid change in the same residue is pathogenic; PP3) 12 *in silico* analyses predicted a deleterious rule of the variant; and PP2) other missense mutations in VCP gene have been reported as pathogenetic. This variant to our knowledge was described for the first time and the impact on the VCP activity and expression is not known. However, the *in silico* analysis predicted a deleterious role on VCP protein. Moreover, the VCP protein alignment across different species demonstrates that this substitution affects an evolutionarily highly conserved amino acid (Figure 4).

## Discussion

Inclusion body myopathy (IBM) with Paget’s disease of bone (PDB) and/or frontotemporal dementia (FTD) (IBMPFD) was described as rare autosomal dominant disorder due to mutations in the *VCP* gene (Saracino et al., 2018). It has been estimated that IBM, PDB, and FTD are present in 80–90, 43–51, and 30% of the mutated individuals, respectively (Fanganiello et al., 2011). However, it has been shown that in 3% of patients FTD is the only feature due to *VCP* mutations (Al-Obeidi et al., 2018). This phenotypic variability of *VCP*-related disease is due to the VCP protein involvement in multiple cellular activities (Watts et al., 2004; Meyer & Weihl, 2014). To date, the majority of *VCP* mutations cluster in exon 3 and 5, which are in N terminus domains of protein involved in binding cofactors and ubiquitylated protein substrates (Niwa et al., 2012; Wang et al., 2004). In this study, we reported a novel missense heterozygous variant c.1184A > C (*p*.D395A) in exon 10 of *VCP* gene identified in three patients, belonging to a Calabrian family of southern Italy. Their clinical and neurobehavioral profile were characterized by insidious early onset and gradual progression of cognitive impairment, apathy, irritability, inattention, loss of insight, emotional blunting, and progressive speech–language impairment until mutism. All patients also manifested bulimia, wandering, and epileptic seizures. The whole picture was consistent with behavioral variant of FTD (bvFTD) (Rascovsky et al., 2011). Unlike many reports about *VCP*-disease, the three mutated siblings have no familial history of IBM and PDB. After 10 years from disease onset, their phenotypes become similar to each other and not characterized by any clinical signs and symptoms compatible with musculoskeletal disorders in agreement with the patient described by Saracino et al. (2018) who carried a c.353C > T, *p*.Pro118Leu mutation. Unlike the mean age of onset of bvFTD (Bang et al., 2015), dementia was diagnosed earlier at mean age of 40 years (40 ± 4.3), whereas degeneration and atrophy were associated with changes in personality and progressive loss of language as in *VCP*-related disease cases. While usual clinical phenotype of *VCP* mutation carriers is characterized by neuropsychiatric manifestations compatible with bvFTD, cases of semantic variant primary progressive aphasia (svPPA) and dementia of the Alzheimer type have also been reported (Mehta et al., 2013). These evidences are in agreement with cognitive deterioration showed by patient II-2 with memory impairment and spatial orientation disorders that progress towards behavioral disorders. In literature cases, disease duration is about 19 years after the onset of IBM and PDB but only 6 years after dementia onset (Mehta et al., 2013). In sum, the peculiarity of our family is represented by the fact that the expression of bvFTD symptoms marks an early onset of the disease that is not associated with clinical and instrumental findings attributable to IBM and PDB even during its evolution. As already discussed in the results, the D395A variant may be considered pathogenic since it affects an aspartate residue positioned in the D1 domain where another mutation (*p*.N387H) was described as pathogenic with negative effects on ATPase activity (Niwa et al., 2012). In addition, Darwich et al. (2020) reported a different amino acid change (c.1194A > G) in the same site that caused dementia with neuronal vacuoles and neurofibrillary tangles. Close to D395A substitution, Watts et al. (2007) reported a change in position 387 (N387H) in a Poland family with IBM and dementia. They found that this mutation, like other mutated residues, clustered in the region of the interface between the CDC48 N-domain with the D1-ATPase domain. This in turn changes the N-terminal conformation, potentially affecting binding interactions within this domain. We speculate that D395A mutation would affect the potential ligand binding properties of this region rather than the catalytic site of the D1 domain. However, functional studies are needed to finally confirm the pathogenicity and to validate the biological impact of this mutation.

In conclusion, our report confirms the high degree of heterogeneity of *VCP*-disease enriching the genotypic spectrum of *VCP* gene with the novel D395A mutation and expands the clinical heterogeneity of *VCP* mutation carriers, showing an incomplete penetrance of the disorder and a heterogeneous picture in different families carrying the same mutation (Van der Zee et al., 2009). This study suggests that a *VCP* analysis should be considered for the genetic screening of familial bvFTD with an early onset also in absence of clinical sings of IBM or PDB.

## Data Availability

The datasets for this article are not publicly available due to concerns regarding participant/patient anonymity. Requests to access the datasets should be directed to the corresponding authors.
